# A clinician-nurse model to reduce early mortality and increase clinic retention among high-risk HIV-infected patients initiating combination antiretroviral treatment

**DOI:** 10.1186/1758-2652-15-7

**Published:** 2012-02-17

**Authors:** Paula Braitstein, Abraham Siika, Joseph Hogan, Rose Kosgei, Edwin Sang, John Sidle, Kara Wools-Kaloustian, Alfred Keter, Joseph Mamlin, Sylvester Kimaiyo

**Affiliations:** 1 Indiana University, School of Medicine, 1001 West 10th Street, OPW-M200, Indianapolis, IN 46202, USA; 2 Moi University, School of Medicine, Eldoret, Kenya; 3 Academic Model Providing Access to Healthcare (AMPATH), Eldoret, Kenya; 4 Regenstrief Institute, Inc., Indianapolis, USA; 5 Brown University, Department of Biostatistics, Providence, USA

**Keywords:** Antiretrovirals, Mortality, Losses to follow up, Adherence, Models of care, Africa

## Abstract

**Background:**

In resource-poor settings, mortality is at its highest during the first 3 months after combination antiretroviral treatment (cART) initiation. A clear predictor of mortality during this period is having a low CD4 count at the time of treatment initiation. The objective of this study was to evaluate the effect on survival and clinic retention of a nurse-based rapid assessment clinic for high-risk individuals initiating cART in a resource-constrained setting.

**Methods:**

The USAID-AMPATH Partnership has enrolled more than 140,000 patients at 25 clinics throughout western Kenya. High Risk Express Care (HREC) provides weekly or bi-weekly rapid contacts with nurses for individuals initiating cART with CD4 counts of ≤100 cells/mm^3^. All HIV-infected individuals aged 14 years or older initiating cART with CD4 counts of ≤100 cells/mm^3 ^were eligible for enrolment into HREC and for analysis. Adjusted hazard ratios (AHRs) control for potential confounding using propensity score methods.

**Results:**

Between March 2007 and March 2009, 4,958 patients initiated cART with CD4 counts of ≤100 cells/mm^3^. After adjusting for age, sex, CD4 count, use of cotrimoxazole, treatment for tuberculosis, travel time to clinic and type of clinic, individuals in HREC had reduced mortality (AHR: 0.59; 95% confidence interval: 0.45-0.77), and reduced loss to follow up (AHR: 0.62; 95% CI: 0.55-0.70) compared with individuals in routine care. Overall, patients in HREC were much more likely to be alive and in care after a median of nearly 11 months of follow up (AHR: 0.62; 95% CI: 0.57-0.67).

**Conclusions:**

Frequent monitoring by dedicated nurses in the early months of cART can significantly reduce mortality and loss to follow up among high-risk patients initiating treatment in resource-constrained settings.

## Background

Combination antiretroviral treatment (cART) has proven itself to be an effective therapeutic mechanism for suppressing viral replication and enabling reconstitution of the immune system, thus allowing patients to recover and live with HIV disease as a chronic illness [1-3]. If adherence to the medications is high, severe immune-suppression is not present at cART initiation, and no significant co-morbidities, such as hepatitis C infection, exist, projections suggest that people living with HIV/AIDS have greatly improved long-term prognosis [4]. Despite the proven effectiveness of cART in low-income countries [5-9], mortality rates among patients in these settings are higher than those seen in high-income environments [10].

In resource-poor settings, mortality is at its highest during the first 3 months after cART initiation [9-12]. It is at least four times higher than rates in high-income countries in the first month of treatment [10]. Why mortality is at its highest during this period has been the subject of much debate and speculation. Reasons for these differences have been attributed to the non-use of cotrimoxazole prophylaxis [13,14], tuberculosis-associated immune reconstitution inflammatory syndrome (IRIS) [15-17], IRIS due to other opportunistic infections [18], and hepatotoxicity related to antiretroviral agents [19]. A consistently clear predictor of mortality during this period is having a low CD4 count at the time of treatment initiation [10,20].

Recent estimates by the World Health Organization (WHO) indicate that although 6.7 million individuals in low- and middle-income settings are receiving cART, this represents only 47% coverage of individuals who are in clinical need [21]. The massive scale up of HIV care and treatment programmes has required enormous investments, and still there is a substantial unmet need. Thus, the challenge presented to HIV care programmes operating in resource-poor settings is how to continue scaling up while simultaneously improving the outcomes of those enrolling in treatment programmes. As such, novel models of care, such as task shifting [22-24], which increase healthcare efficiency and improve patient outcomes, clearly need to be designed and tested.

Here, we describe the impact of a nurse-clinician approach [25] on mortality and patient retention among severely immune-suppressed HIV-infected adults initiating cART within a large multi-centre HIV/AIDS care and treatment programme in western Kenya.

## Methods

### Study design

This was a retrospective analysis of prospectively collected routine clinical data. The study was approved by the Indiana University School of Medicine Institutional Review Board and the Moi University School of Medicine Institutional Review and Ethics Committee.

### The programme

The Academic Model Providing Access to Healthcare (AMPATH) was initiated in 2001 as a joint partnership between Moi University School of Medicine in Kenya, the Indiana University School of Medicine, and the Moi Teaching and Referral Hospital. The USAID-AMPATH Partnership was initiated in 2004 when AMPATH received ongoing funding through the United States Agency for International Development (USAID) and the United States Presidential Emergency Plan for AIDS Relief (PEPFAR). The initial goal of AMPATH was to establish an HIV care system to serve the needs of both urban and rural patients and to assess the barriers to and outcomes of antiretroviral therapy. Details of the development of this programme have been described in detail elsewhere [26].

The first urban and rural HIV clinics were opened in November 2001. Since then, the programme has enrolled more than 140,000 HIV-infected adults and children in 25 Ministry of Health (MOH) facilities and numerous satellite clinics in western Kenya (data for satellite clinics are incorporated into their "parent" clinic). Although located within the MOH facilities, the AMPATH clinics are dedicated to HIV and HIV/TB care, treatment and support. All HIV- and tuberculosis-related care and treatment are provided free at the point of care.

#### Clinical procedures: express care and routine care

The HIV clinical care protocols used by the USAID-AMPATH Partnership are consistent with those recommended by WHO and have been described in detail elsewhere [27]. Briefly, the Routine Care protocol for patients receiving cART is that patients are seen by the clinician (clinical officer or physician) 2 weeks after initiating treatment, and then monthly thereafter. Those who have not initiated cART return every 1 to 3 months depending on their clinical status and co-morbidities.

During these visits patients are seen by multiple care providers, including nurses, clinicians, pharmacy technicians, nutritionists, peer outreach workers and social workers. For new patients, clinical contact begins at registration, followed by the nurse who checks vital signs. The patient also sees a peer outreach worker for documentation of locator information, then goes on to see the doctor/clinical officer. Returning patients go directly to the nurse and then follow a course similar to that established for new patients. All patients newly initiated on cART who miss a scheduled clinic visit trigger an outreach attempt within 24 h, through either a telephone contact or home visit conducted by trained peers. Standard first-line antiretroviral regimens used are either nevirapine-based or efavirenz-based [28].

Beginning in March 2007, the High Risk Express Care (HREC) programme was implemented in a step-wise fashion in USAID-AMPATH Partnership clinics. As of May 2008, HREC had been rolled out to all parent clinics. The selection of clinics to pilot the programme was based primarily on space availability, patient volume and clinic congestion, and the general capacity of healthcare personnel to pilot a new programme. Once a clinic had implemented HREC, all patients meeting the criteria for HREC were eligible for referral to the programme. The criteria for referral to HREC are having a CD4 count of 100 cells/mm^3 ^or less and initiating cART.

Once a patient is identified as eligible for HREC, the clinical officer or physician prescribes a two-week supply of cART and then refers the patient to HREC (within the same clinic location). The patient is seen by a clinical officer or physician 2 weeks after antiretroviral initiation and then monthly. The HREC nurse is then responsible for interim weekly visits either physically or by telephone for a period of 3 months. The patient is seen monthly by a clinical officer or physician. In HREC, returning patients do not queue in the waiting bay or go through the nursing station, clinician room, pharmacy or any other referral points within the clinic. Instead, such patients go directly to the "Express Care room", which provides "one-stop care". The HREC nurse maintains a list of scheduled return visits, and if a patient misses a clinic appointment, the outreach team is activated as per routine protocol.

The HREC visit for the high-risk patients is focused on identifying co-morbidities and complications of cART and reinforcing medication adherence. The nurse asks about adherence to medication by asking whether the patient has missed any of his/her medications in the previous 7 days and then conducting a pill count. If the patient is not perfectly adherent, he or she is referred to the clinical officer or physician. The nurse reviews a symptom checklist (new cough, breathlessness, rash, jaundiced eyes, vomiting, diarrhoea, severe headache, fever or "any other problem that you feel you need a doctor for") and measures temperature and transcutaneous oxygen saturation. If the patient reports any symptoms or meets the pre-set threshold for either temperature (≥37.2°Celsius) or oxygen saturation (O_2 _≤ 93%), the patient is referred immediately to the clinical officer or physician. The nurse does not dispense drugs during an HREC visit as these are prescribed during monthly clinical officer or physician visits. A summary of similarities and differences between HREC and Routine Care can be found in Table 1.

**Table 1:** Summary of programme characteristics in High Risk Express Care (HREC) versus Routine Care

	Routine care	HREC
Initial clinical assessment	Yes	No
Prescription of antiretrovirals	Yes	No
Interim clinical assessments including weight and vital signs	Yes, monthly by clinical officer	Yes, weekly by nurse and monthly by clinical officer
Adherence monitoring	Yes, monthly	Yes, weekly
Defaulter tracing	Yes, within 24 h	Yes, within 24 h

### Data collection

Clinicians complete standardized forms capturing demographic, clinical and pharmacologic information at each patient visit. These data are then hand-entered into the AMPATH Medical Record System, a secure computerized database designed for clinical management, with data entry validated by random review of 10% of the forms entered [29]. At the time of registration, patients are provided with a unique identifying number. For this study, all data were stripped of identifying information prior to analysis.

### Study population

The analysis included all patients aged 14 years or older who were initiating cART with CD4 counts less than or equal to 100 cells/mm^3 ^in one of the USAID-AMPATH clinics from March 2007 until March 2009.

### Outcomes, explanatory variables and confounders

The primary goal of the HREC system is to prevent mortality during the first 3 months following cART initiation. Therefore, the primary outcome for this analysis was all-cause mortality. Two secondary outcomes were analyzed: loss to follow up (LTFU), defined as being absent from the clinic for at least 3 months with no information regarding vital status, and a composite outcome defined as LTFU or death. The rationale for the composite outcome is that loss to follow up in such a high-risk population is likely to mean that a patient has died, even if the death has not yet been reported to the clinic [10,30-34]. Analysis of the composite outcome can be viewed as a sensitivity analysis for the mortality rate as it provides an upper bound of the mortality estimate.

The primary explanatory variable is being in the HREC programme (versus remaining in Routine Care). Our analyses quantify the effect of HREC on mortality, loss to follow up, and the composite outcome of death or loss to follow up using crude and adjusted hazard rate (HR) ratios.

Our adjusted HR ratios control for the following potential confounding variables, all measured at time of cART initiation and selected *a priori *based on their potential to independently affect changes in risk of mortality and/or loss to follow up: CD4 count (analyzed as a continuous variable); receipt of treatment for tuberculosis at the time of cART initiation (yes/no); receipt of cotrimoxazole or dapsone prophylaxis within 28 days of cART initiation (yes/no) [13,17]; travel time to clinic (dichotomized as up to one hour vs. more than one hour); type of clinic (referral hospital, district or sub-district hospital, or rural health centre); age; and sex (male/female). CD4 and age are centred at their mean values. WHO clinical stage was not included in the final model because: a) it was non-significantly associated with the outcomes of interest in bivariable analyses; and b) there was missing data in one of the two groups.

### Analysis

We included all eligible patients and categorized them as having been enrolled into HREC at initiation of cART or having remained in Routine Care. The distributions of the time to event outcomes are summarized using Kaplan-Meier curves. Event times and censoring times are defined as follows: for analysis of mortality, the event time is the date of death; others are censored at the time of their last clinic visit. For the analysis of loss to follow up, patients were defined as lost if they had not returned to clinic for more than three consecutive months; for these patients, the event time is the 90^th ^day following the most recent visit to the clinic.

Individuals whose reported follow-up time is zero days had 1 day added for the purpose of analysis. Those who died were censored on their death date, and patients whose most recent clinic was less than 90 days prior to the close of the database were censored at the date of their last clinic visit. For the loss to follow up analysis, the earliest possible event time is 90 days after the initiation of cART; hence our tests and regression models use a start time of day 90. Finally, for the composite outcome, the event time is the earliest of death date or loss to follow up date, where the LTFU date is defined as we have explained.

The adjusted HR ratios control for potential confounding variables using inverse weighting by the treatment propensity score method [35]. For each individual, the propensity score is the probability of receiving HREC as a function of individual level characteristics. The propensity score, denoted by *p*(*x*), is estimated by fitting a logistic regression model of HREC status (yes/no) on potential confounding variables *x*. The propensity score model is checked for lack of fit using the Hosmer-Lemeshow goodness-of-fit test [36]. The adjusted HR ratios are calculated by fitting a weighted, stratified proportional hazards regression of the event time on HREC status. The weights are proportional to 1/p(x) for those who receive HREC, and to 1/{1 - p(x)} for those who do not. Following Hernan et al. [27], stabilized weights were used in the estimation (full details are available upon request). The stratification variable is clinic type. Robust standard errors are used to account for clustering by clinic and for correlation induced by the use of inverse probability weights.

Because the propensity scores represent the probability of receiving HREC as a function of individual-level covariates (listed here), the weight for each individual is inversely proportional to the (estimated) probability of his or her actual HREC status. The weighted sample can therefore be viewed as one where differential selection into HREC attributable to *x *has been eliminated; hence, to the extent that *x *contains all relevant confounders, the adjusted HR is equivalent to the exposure effect from a marginal structural proportional hazards model and can be interpreted as the causal effect of HREC on the event of interest [37,38].

All analyses were done using STATA Version SE/10 (College Station, Texas, USA).

## Results

There were 4,958 patients aged 14 years or older with CD4 counts of ≤100 cells/mm^3 ^who initiated cART at one of the USAID-AMPATH clinics during the study period. Of these, 635 were enrolled into HREC. Reasons why patients were not enrolled into HREC included that the HREC programme had not yet been rolled out to a particular clinic, that the patient lived too far to allow them to attend clinic weekly or bi-weekly, and lack of available space in the clinic for expansion of the HREC programme.

As summarized in Table 2 patients in Routine Care and HREC were similar with regard to gender distribution and age: 40% male with a median age of approximately 36 years. There were no significant differences between the groups with regard to baseline CD4 count or proportion receiving treatment for tuberculosis. Patients in HREC were slightly less likely to be WHO Stage III/IV at cART initiation (66% vs. 69%) and to have to travel at least 1 h to clinic (71% vs. 77%). They were more likely to be attending an urban clinic (61% vs. 52%), and using cotrimoxazole or dapsone prophylaxis at cART initiation (100% vs. 95%). The median follow-up time was 318 days (interquartile range 147-533).

**Table 2:** Socio-demographic and clinical characteristics of patients in High Risk Express Care (HREC) versus Routine Care

Variable	HREC n = 635	Routine care n = 4323	p value
**Male gender**	256 (40%)	1749 (41%)	0.945
**Median (IQR) age**	35.8 (30.5-42.4)	36.7 (30.6-43.1)	0.097
**Median (IQR) CD4 at initiation of cART**	46 (20-72)	46 (20-74)	0.908
**WHO Clinical Stage III/IV at initiation of cART**	418 (66%)	2966 (69%)	0.031
***Missing***	0 (0%)	89 (2.1%)	
**On TB treatment at cART initiation**	204 (32%)	1514 (35%)	0.152
**Clinic type**			
**Referral hospital**	217 (34%)	903 (21%)	< 0.001
**District and sub-district** **hospitals**	221 (35%)	2107 (49%)	
**Rural health centres**	197 (31%)	1313 (30%)	
**Use of cotrimoxazole or dapsone within 28 days of cART initiation**	632 (100%)	4097 (95%)	< 0.001
**Travel > 1 h to clinic**	452 (71%)	3318 (77%)	0.002

N.B. The only variable for which there were missing data was WHO Stage, as described in the table. This variable was not included in the propensity score model as it was not a significant predictor of the outcomes of interest

**Figure 1:**
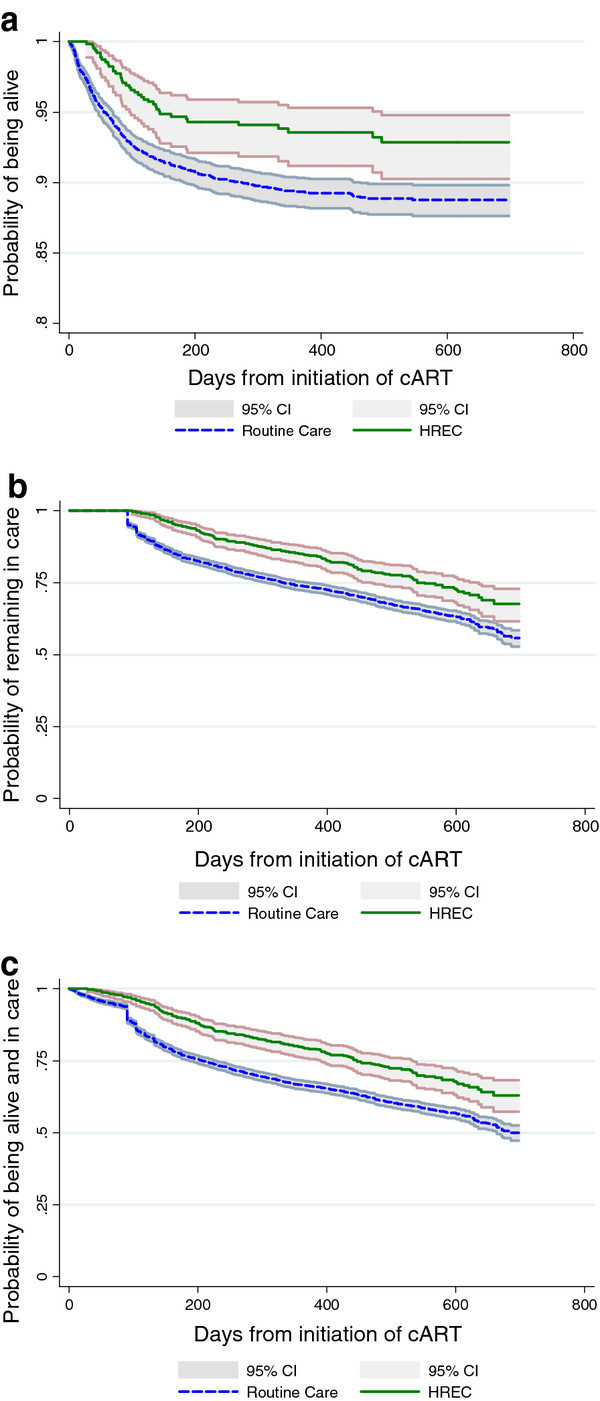
**Kaplan-Meier curves demonstrating the effect of the High Risk Express Care compared with Routine Care among all high-risk patients on: a) their probability of survival; b) their probability of remaining in care (i.e., loss to follow up); and c) their probability of remaining alive and in care (n = 4958). a Probability of remaining alive after cART initiation. b Probability of remaining in care after cART initiation. c Probability of remaining alive and in care after cART**.

There were 426 deaths among the study population: 39 in HREC and 387 in Routine Care. The crude incidence rate of death during the follow-up period was 5.7 per 100 person-years in HREC compared with 10.6 per 100 person-years in Routine Care (incidence rate ratio, IRR: 0.54; 95% confidence interval, CI: 0.38-0.75) (Figure 1a). After adjustment for potential confounders, the HREC programme was associated with a 40% reduced risk of death (adjusted HR: 0.59; 95% CI: 0.45-0.77).

There were also 1,299 patients lost to follow up during the same period, including 134 in HREC and 1,165 in Routine Care. The crude incidence rate of LTFU among HREC was 18.7 per 100 person-years versus 29.7 in Routine Care (IRR: 0.63; 95% CI: 0.52-0.76) (Figure 1b). After adjustment, patients in HREC were also much less likely to become lost to follow up (AHR 0.62; 95% CI: 0.55-0.70).

When we assessed the combined endpoint of LTFU and death, there were 1,725 events in 4639.5 person-years of follow up, for a crude incidence rate of 24.2 per 100 person-years in HREC versus 39.5 in Routine Care (IRR: 0.61; 95% CI: 0.52-0.72) (Figure 1c). Overall, the HREC patients were much more likely to be alive and in care after starting cART by the end of the study follow-up period (AHR 0.62; 95% CI: 0.57-0.67) (Table 3).

**Table 3:** Adjusted Hazard Ratios (HR)* and 95% Confidence Intervals (CI) of the effect of High Risk Express Care (HREC) vs. Routine Care on: a) Death, b) Lost to Follow-up (LTFU), and c) Death or LTFU (combined endpoint) following cART initiation

Effect of high risk express care on eligible patients	Death	Loss to follow-up	Death or
			Loss to follow-up
	N events: 426	N events: 1299	N events: 1725
Unadjusted HR	0.60 (0.48-0.74)	0.63 (0.56-0.71)	0.63 (0.58-0.69)
(95% CI)	Robust Std. Err.: 0.06	Robust Std. Err.: 0.04	Robust Std. Err.: 0.03

Adjusted HR	0.59 (0.45-0.77)	0.62 (0.55-0.70)	0.62 (0.57-0.67)
(95% CI)	Robust Std. Err.: 0.08	Robust Std. Err.: 0.04	Robust Std. Err: 0.03

*Adjusted using propensity scores for clinic, the use of cotrimoxazole or dapsone, CD4 count closest to cART initiation, receiving treatment for tuberculosis,, gender, age, clinic type (Referral Hospital, Sub-District and District Hospitals, and Rural Health Centres), and travel time to clinic.

## Discussion

The first few months following initiation of cART is a critical time for severely immune-suppressed HIV-infected patients. These data suggest that more frequent monitoring of patients in the early months by a dedicated nurse can significantly improve survival and retention in care among these high-risk patients and improve their retention in care. Although further evaluation is needed, this intervention may be relatively easy to implement in other resource-constrained environments.

To our knowledge, the concept of frequent nurse-based rapid assessments is among the few interventions other than cotrimoxazole prophylaxis that has been associated with a profound reduction in early mortality among high-risk HIV-infected patients initiating cART in low-income settings [13,14]. We believe the effects of HREC are a combination of the rapid *and *frequent assessments. Rapid because this makes accessing healthcare more accessible to patients (by not having to wait as long and by not having to spend as much time in the clinic); frequent because it makes it more likely that early warning symptoms (e.g., fever, rash) can be identified within days, as opposed to weeks, of their onset. If, for example, the monthly standard of care visits were simply made more rapid, such symptoms as fever or rash would go unattended for potentially weeks, thereby increasing the risk of full-blown immune-reconstitution disease, more severe toxicities, etc.

We also postulate that more frequent monitoring is effective at improving early patient outcomes through both direct and indirect mechanisms. For example, earlier identification of the signs and symptoms of drug toxicity, opportunistic infections and immune reconstitution syndrome likely leads to earlier interventions to address these issues; thus patients should experience a direct survival advantage in the short-term.

Indirectly, HREC may have improved adherence to cART and thus improved short-term outcomes. Adherence may be improved in the HREC population because of the weekly contact, reminders and supports; as a result of improved adherence, patients will be more likely to experience complete virologic suppression, have improved immune-response, and be less likely to develop resistance, therefore indirectly contributing to survival benefits over the short and long term. HREC may have improved retention by enabling patients to be seen quickly without having to wait in long queues and without having to pass through multiple stations (i.e., spending much of the day at the clinic), thereby making their healthcare more accessible to them.

There are some additional costs associated with HREC. For example, there are added direct costs to patients arising from increased transportation required to and from the clinic. They may also have to miss more work because of more frequent clinic visits, translating into increased opportunity costs. The nurses hired to work on HREC were hired explicitly for that purpose, and this certainly adds to the overall programme expense on personnel. Whether these additional costs and expenses are justified when weighed against the costs associated with increased morbidity, mortality and loss to follow up is the subject for a detailed cost-effectiveness analysis that is beyond the scope of the present evaluation.

There are two key strengths to these findings. First, the standard of care in AMPATH is already to provide cotrimoxazole or dapsone until a patient's CD4 count is above 200 cells/mm^3^. These data have therefore been able to assess the effect of more frequent monitoring in a setting where the vast majority of patients were already receiving cotrimoxazole or dapsone. Second, the effect of HREC was strong across three different versions of the primary outcome, suggesting a robust effect. By evaluating the impact of HREC on both mortality and LTFU, we took account of the two crucial factors determining the success of an HIV treatment programme: keeping patients alive and in care. Moreover, we used statistical methods that appropriately and robustly adjusted for measured confounders.

There are also limitations to this analysis. First, the choice of clinics in which HREC was rolled out to first may have created a variety of potential biases in our analysis related to a possible higher quality of care offered at those clinics. For example, this may have created some selection bias (improved patient outcomes at those clinics irrespective of HREC, e.g., because of lower patient volumes, or higher functioning staff), and ascertainment bias (better ascertainment of death and other clinical outcomes at the higher functioning clinics). Similarly, although all patients were eligible to be enrolled into HREC, only a small proportion were enrolled. If the providers who were more likely to refer patients to HREC were also the ones more likely to be current with clinical protocols (i.e., to prescribe cotrimoxazole) and/or more likely to be better clinicians, those patients referred by them may have been more likely to have better outcomes anyway.

However, for those patients referred, it was the HREC nurse who had the majority of clinical contact with them, thereby reducing any potential provider bias on the part of the referring clinician. Similarly, a slightly smaller proportion of patients enrolled into HREC were WHO Stage III/IV at treatment initiation. Although these issues may have biased the findings favourably towards HREC, the use of propensity score methods helps to overcome this possible bias because weighting in inverse proportion to the treatment propensity score creates a pseudo-sample wherein allocation to HREC is independent of the confounders that have been included in the propensity score. Hence the weighted dataset can be analyzed as if the group allocation were random. A limitation of this method is that there may be non-random allocation to HREC based on unmeasured factors to the extent that receipt of HREC depends on unmeasured factors. We acknowledge that residual bias may therefore remain due to potential unmeasured confounders, including adherence to cART (not accounted for in this analysis due to unreliability of the data).

## Conclusions

In conclusion, we have demonstrated that weekly rapid assessments by nurses, either by phone or in person, with immediate referrals to clinical officers or physicians if needed, can significantly improve survival among high-risk HIV-infected patients initiating cART in a sub-Saharan African setting. Although the cost effectiveness of the Express Care model needs to be thoroughly evaluated, our experience and findings suggest that this may be an innovative way of increasing patient volume, improving the quality of care, and greatly improving patient outcomes in the short term.

## Competing interests

The authors declare that they have no competing interests.

## Authors' contributions

PB was primarily responsible for the writing of the manuscript. AS, RK, JS, JM, and SK are practicing physicians in Kenya who developed the Express Care programme and contributed significantly to the generation of hypotheses and interpretation of results for the manuscript. JH is the biostatistician on record for this analysis and provided significant input and technical assistance in the methodological approaches used. AK was the analyst for the study. KWK critically reviewed the manuscript for issues of design and interpretation. ES was the data manager responsible for providing and overseeing the data. All authors have read and approved the final version of this manuscript.
